# Capturing Acquired Wisdom, Enabling Healthful Aging, and Building Multinational Partnerships Through Senior Global Health Mentorship

**DOI:** 10.9745/GHSP-D-20-00108

**Published:** 2020-12-23

**Authors:** C. Norman Coleman, John E. Wong, Eugenia Wendling, Mary Gospodarowicz, Donna O’Brien, Taofeeq Abdallah Ige, Simeon Chinedu Aruah, David A. Pistenmaa, Ugo Amaldi, Onyi-Onyinye Balogun, Harmar D. Brereton, Silvia Formenti, Kristen Schroeder, Nelson Chao, Surbhi Grover, Stephen M. Hahn, James Metz, Lawrence Roth, Manjit Dosanjh

**Affiliations:** a International Cancer Expert Corps, Washington, DC, USA.; b National University Health System, Singapore.; c Princess Margaret Cancer Center, University of Toronto, Toronto, Canada.; d National Hospital, Abuja, Nigeria.; e Abuja College of Medicine, Nigeria.; f Radiation Research Program, National Cancer Institute, Washington, DC, USA.; g TERA Foundation, Novara, Italy.; h Department of Radiation Oncology, Weill-Cornell Medical Center, New York City, NY, USA.; i Duke University School of Medicine, Durham, NC, USA.; j Bugando Cancer Center, Mwanza, Tanzania.; k University of Pennsylvania, Philadelphia, PA, USA.; l University of Botswana & Princess Marina Hospital, Gaborone, Botswana.; m European Organization for Nuclear Research (CERN), Geneva, Switzerland.

## Abstract

The undeniable benefit of mentorship by experience senior mentors can meaningfully increase the breadth of their experience and contributions to society as well as address the dire inequality in global health. This model captures wisdom lost to retirement, enables opportunities for purposeful lifespan, underpins sustainable health care systems, and has the potential for building multinational partnerships.

## Key Messages

- Capturing the acquired wisdom and experience of mentors in global health offers a capstone for their careers and provides a purposeful healthspan for these professionals to continue to be engaged in meaningful work while leveraging their expertise to solve challenging health care problems.
- Senior professionals can mentor early career leaders to help them balance their professional commitments, interest in global health, and development of needed skills, such as understanding the nuances of cultural competence and adapting solutions to different environments.
- Institutional leaders, particularly in academic medical centers, recognize the importance of global engagement vis-à-vis their educational mission and for recruiting and retaining faculty and can benefit economically and programmatically from supporting experienced senior faculty or retirees to support these efforts.
- Program builders should include the opportunity for altruistic human service as an integral part of a career and highlight that they can access senior mentors and retirees who provide world-class expertise and mentorship at “volunteer prices.”

## INTRODUCTION

An opportunity to have a substantial impact on multiple challenging societal problems exists in simultaneously addressing the following: (a) the urgent need for sustainable health care; (b) the importance of mentorship in enabling the emergence of new generations of leaders; (c) the essential need for cross-cultural competency^1^ to address global crises through problem solving across societal boundaries; and (d) options for continued productivity by the increasing number of older people. Sustainable health care needs to build on cancer care, which requires urgent intervention and encompasses noncommunicable and infectious diseases in low- and middle-income countries (LMICs) and geographically isolated populations in high-income countries (HICs). Capacity building to meet the cancer care gap, which builds sustainable infrastructure for overall health care and economic development, can be done through twinning programs that engage senior health care professionals in meaningful mentoring roles. As the capstone of a career, these professionals thereby create next-generation leaders within LMICs and their own institutions. This article addresses such opportunities available for individuals in the latter part of their careers including postretirement done either as a continuation of their role as career-long mentors or as a new challenge to be met with their lifelong experience. The expanding and branching tree of mentors to mentees enables a career path in global health and geometric growth to fill in the current enormous capacity gap.

## PURPOSEFUL AGING

The challenges facing society regarding the aging of the population are complex. Concepts that have emerged over the past few years to address these challenges include that of “healthspan—the period of life spent in good health, free from the chronic diseases and disabilities of aging”^2^ and the benefit to purpose in life (PIL) for improved health outcomes. Musich et al.^3^ noted:

*PIL is strongly associated with improved mental and physical health outcomes among older adults. Thus, interventions to improve and/or maintain higher levels of PIL over time may promote successful aging.*

This article describes opportunities for professionals to utilize their time and expertise to address the unacceptable gap in cancer care in underserved communities in LMICs and in geographically isolated areas in HICs. Regardless of whether this type of activity promotes longer or healthier lives,^4^ it captures expertise that is all too often lost and thereby transfers experience and wisdom to younger generations.

## UNIQUE APPROACH TO THE CHALLENGE OF GLOBAL HEALTH CARE

Cancer and other noncommunicable diseases (NCDs) represent an increasing share of the global burden of disease in both resource-rich and -poor countries, primarily due to aging, industrialization, sedentary lifestyle, pollution, diet, and the successful approaches to and investment in tackling infectious diseases.^5^ ^,^ ^6^ Indeed, addressing the full spectrum of cancer care—prevention, screening, diagnosis, treatment, and long-term follow-up—requires addressing the other major NCDs, such as respiratory, cardiovascular, and metabolic diseases, as well as infectious diseases involved in cancer etiology and those related to treatment.^7^ LMICs lack infrastructure, resources, and expertise to address this problem. For example, the workforce shortfall in LMICs is highlighted by the Lancet Oncology Commission’s Global Task Force on Radiotherapy for Cancer Control of the Union for International Cancer Control.^8^ Using current staffing models, this report estimates that, by 2035, an additional 30,000 radiation oncologists and over 100,000 technical personnel, as well as clinical support and research staff, will be needed worldwide. The essential health care system expertise and infrastructure needs and the benefits that would be derived from filling these health care gaps make this a formidable and compelling challenge.

Mentorship is recognized as an important element in health care training.^9^ Leveraging the expertise and mentorship of senior experts can alleviate this shortfall. Fortunately, because many people are living well past the historical retirement age of 60–70 years, the upward shift of the population age distribution offers a golden opportunity to capture global wisdom, address inequalities, and leverage mentorship and innovative technology to enable sustainable improvement of global health. How best to remain a useful contributor to one’s community and society is a predictable challenge, especially for professionals who have developed the highly sophisticated skill sets required for health care and desire to continue to use their professional knowledge meaningfully. These senior members of a profession also have perspective on the current economic situation in health care, medical and scientific knowledge, and societal trends, as well as broad hands-on patient engagement skills that are particularly relevant in health care in which training and advancement follow a skill-based apprenticeship model.

How best to remain a useful contributor to one’s community and society is a predictable challenge, especially for highly skilled professionals who desire to continue to meaningfully use their knowledge.

## PERSON-TO-PERSON CONNECTIVITY AS A SOLUTION SET

The unprecedented scope of the problems facing humanity today, including climate change, wealth disparities, xenophobia and related terrorism, potential for pandemics, and depletion of natural resources, among others, absolutely requires problem solving across cultures and boundaries. The necessary trusted partnerships/friendships and cultural competence can come from career-long diplomats, altruists, and science-based collaborations, bringing in opportunities for groups such as Peace Corps volunteers,^10^ professional societies, and nongovernmental organizations. Such organizations span generations, from the eager student to the individuals with decades of experience. The lifelong acquired wisdom of the latter is often lost to retirement, but it is necessary for effective transitions and the transmission of knowledge. Helping those early in their career to visualize a career path in altruistic service can be a powerful motivator and reinforce their own career choices.

A novel approach to address the health care workforce shortfall is the working mentorship model of the International Cancer Expert Corps (ICEC).^11^ It draws on a wide breadth of partners and includes the following:

- A collaborative multi-institutional and multinational organization with opportunities for a broad spectrum of experts, who are needed to build an effective health care enterprise to optimize resource utilization and facilitate the transfer of professional and technologic experience and expertise^12^
- Assignments in established and emerging twinning partnerships with HIC expert academic centers, professional societies, and private practices mentoring programs in LMICs, thereby offering long-term guided progress as opposed to episodic visits^6^
- Tools and resources to guide mentoring and program-building efforts including standard operating procedures, the detailed metrics in the ICEC 5-Step Progression Plan for Cancer Care,^13^ and formal guidelines for education and training programs for global settings^14^
- Ways to contribute expertise to support volunteer education programs such as Chartrounds’ case conferences for LMIC participants^15^
- Opportunities for mentors to get formal recognition for their contributions, as part of a shared mission, while assisting in the development of a career path in global health

Expertise can come from both people and technology. For health care in developing countries and for developed countries in the future, where rising expenses are a major societal issue, building human and technology expertise together, using the rapidly growing area of artificial intelligence and machine learning, can better utilize human resources. Technology requires appropriate training and support services. The teams providing care in some poorly resourced countries may have access to excellent (highly publicized and often very expensive) equipment, but they may not have the expertise to fully utilize it. This problem is being addressed by ICEC and its LMIC partners,^16^ ^,^ ^17^ Medical Physics for World Benefit,^18^ the International Atomic Energy Agency Division of Human Health,^19^ and academia.

The Figure illustrates the mentorship model for patient-centered cancer care, which encompasses a broad range of expertise including NCDs and infectious diseases. Mentorship includes the continuum of mentors, with senior mentors guiding early- and mid-career mentors from well-resourced programs (hubs) who jointly train and educate mentees and staff within LMICs and geographically isolated regions in HICs (centers), thereby geometrically expanding the system of patient-centered care. Senior expertise, a very expensive component of health care (“Solution shop” of Christensen et al.^20^), can be made available much less expensively with this sustainable volunteer mentorship approach. Sharing knowledge and broad expertise in this manner enhances and expands their value well beyond the one-to-one mentor-mentee relationship. This innovative paradigm captures acquired wisdom, which is often lost following retirement, to benefit society.

Mentorship includes the continuum of mentors, with senior mentors guiding early- and mid-career mentors from well-resourced programs who jointly train and educate mentees within LMICs.

**FIGURE:**
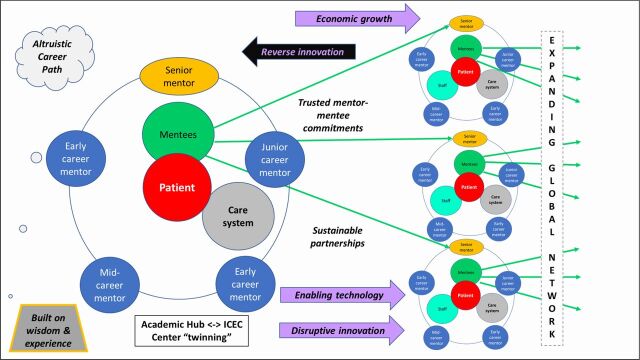
Basic Mentorship Model of Expansion of Expertise for Mentored Patient-Centered Care Abbreviation: ICEC, International Cancer Expert Corps.

**Figure:**
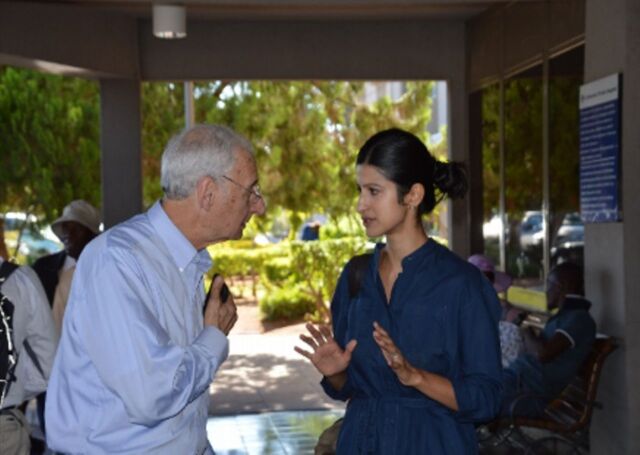
Dr. Norman Coleman and Dr. Surbhi Grover discuss future plans for mentorship and innovative technology in Botswana. ©2019 Manjit Dosanjh

## TWINNING: MENTOR-MENTEE PARTNERSHIPS

The mentorship model illustrated in the Figure works primarily through twinning programs that are collaborative relationships between HIC university departments or private practice programs (hubs) and programs/facilities in an LMIC (centers). The value of mentorship and the exponential impact of transferring experience are apparent in the twinning programs that establish a proper infrastructure for education, training, and mentoring. This capacity-building strategy facilitates the creation of a sustainable platform for the mutual sharing of best practices and learning through information and technology transfer. The ultimate aim is for the centers to achieve the required level of expertise to become hubs for their respective regions. A successful international pioneering example is the King Hussein Cancer Center in Jordan, which is now a regional leader in cancer care. Mentoring at the trainee level is exemplified by the work of the Association of Residents in Radiation Oncology Global Health Initiative.^21^ For problems as large as the gap in global health care that may seem “too hard” to address, specific examples can make the solution less daunting and even an exciting personal challenge.

Because going from concept to operational reality is critical, we include a narrative example of successful mentorship from a mentor and a mentee (Box) (additional examples are included in the Supplement). An important starting point is that even pursuing a sustainable career in global oncology had been a challenge, yet these mentor-mentee teams have opened up this possibility to an emerging generation committed to global health. The mentor-mentee model has already demonstrated success, as shown with examples in Table 1.

**TABLE 1.** International Cancer Expert Corps Mentoring Relationships

**Mentee**	**Mentor**	**Year Started**	**Examples of Achievement**
**National Center of Oncology, Yerevan, Armenia, and Weill Cornell, New York, USA**			
**Onyinye Balogun, MD** Assistant Professor of Radiation Oncology, Weill Cornell	**Silvia Formenti, MD** Chairman Department of Radiation Oncology, Weill Cornell Associate Director of the Meyer Cancer Center and Radiation Oncologist in Chief, New York Presbyterian Hospital **Harmar Brereton, MD** Clinical Professor of Medicine Geisinger Commonwealth School of Medicine and Clinical Assistant Professor of Radiation Oncology, Weill Cornell	2015	Established a training program to facilitate transitioning from 2-dimensional to 3-dimensional treatment planning for treatment of cancer with radiotherapy, with a focus on breast cancer Established education and ongoing training program to ensure proper implementation of image-guided brachytherapy for cervical cancer. Training is delivered through didactic lectures and teleconferences offering patient case discussion and peer review Established the global oncology initiative at Weill Cornell Medicine Established one of the first ICEC twinning programs linking an emerging cancer treatment program in an LMIC with an advanced cancer treatment program in an HIC
**Princess Marina Hospital, Gaborone, Botswana, and University of Pennsylvania, Philadelphia, Pennsylvania, USA**			
**Surbhi Grover, MD, MPH** Assistant Professor of Radiation Oncology, Perelman School of Medicine, University of Pennsylvania University of Botswana & Princess Marina Hospital, Gaborone, Botswana	**Stephen Hahn, MD** FDA Commissioner Former Chair, Department of Radiation Oncology Perelman School of Medicine, University of Pennsylvania **James Metz, MD** Chair, Department of Radiation Oncology, Perelman School of Medicine, University of Pennsylvania	2014	Increased evidence-based care establishing guidelines for the top 10 cancers in Botswana Created an educational exchangeprogram between University of Botswana and University of Pennsylvania Developed research programs between the University of Botswana and University of Pennsylvania Radiation Oncology expanding research capacity at University of Botswana and linking young investigators to international mentors to support research Advanced strategies to reduce stock-outs of chemotherapy and to improve systems to reduce delays in pathology diagnosed through an initiative with the American Society of Clinical Pathology Botswana is now a destination for radiation oncology residents pursuing careers in global health, orchestrated by the Association of Residents in Radiation Oncology
**Mentee**	**Mentor**	**Year Started**	**Examples of Achievement**
**Bugando Cancer Center, Mwanza, Tanzania, and Duke Children’s Hospital & Health Center, Durham, NC, USA**			
**Kristin Schroeder, MD, MPH** Pediatric Hematology-Oncology Specialist, Pediatric Neuro-oncologist Bugando Cancer Center, Mwanza, Tanzania Duke Children's Hospital & Health Center, North Carolina, USA	**Nelson Chao, MD, MBA** Donald D. and Elizabeth G. Cooke Professor Chief, Division of Hematologic Malignancies and Cellular Therapy/BMT Director, Global Cancer, Duke University School of Medicine	2014	Established a patient navigator program that offers education and caregiver guidance throughout the diagnosis and patient treatment Developed a pediatric cancer clinical database to monitor patient outcomes Established a hospital-based cancer registry Fostered a streamlined process to speed cancer diagnosis and access to treatment Implemented standard protocols for care Initiated research programs related to Burkitt lymphoma and retinoblastoma treatment, and impact of psychosocial support Founded the NGO, International Cancer Care and Research Excellence Foundation (iCCARE), a nonprofit whose mission is to give any child diagnosed with cancer the same chance of a cure regardless of where they live Her mentorship of 19 individuals includes 2 Fulbright scholars, 5 masters level students, 1 oncology fellow, 2 nurses, 1 resident, 3 medical students, and 3 undergraduate students
**National Hospital, Abuja, Nigeria, and the European Organization for Nuclear Research (CERN)**			
**Taofeeq Abdallah Ige, PhD** Chief Consultant Physicist and Head of Medical Physics Department, National Hospital, Abuja, Nigeria	**Manjit Dosanjh, PhD** Senior Advisor for Medical Applications, CERN	2017	Established cross-border networking, education, and research projects to enhance the accessibility, effectiveness, and safety related to the use of medical physics and technologies improving treatment techniques and patient outcomes Fostered mentoring relationships between individuals in HICs and LMICs providing access to expert knowledge, guidance, advice, and building collegial relationships Established knowledge- and information-sharing programs utilizing various platforms including WebEx and videoconferencing and attendance at global scientific meetings Facilitated engagement in research programs resulting in co-authorship on scholarly articles published in leading academic journal publications
**Mentee**	**Mentor**	**Year Started**	**Examples of Achievement**
**National Hospital, Abuja, Nigeria, and the International Cancer Expert Corps (ICEC)**			
**Simeon Chinedu Aruah MD, MPH, FWACS** Consultant Radiation and Clinical Oncology National Hospital Abuja, Nigeria Lecturer University of Abuja College of Medicine, Nigeria Head of Department Radiation and Clinical Oncology National Hospital Abuja, Nigeria	**David A. Pistenmaa, MD, PHD, FACR** Chief Scientific Program Director International Cancer Expert Corps Manjit Dosanjh, PhD Senior Advisor for Medical Applications, CERN	2017	Fostered academic growth through co-authorship on publications in top scientific oncology journals Presentation of quality papers in different fora Capitalized on opportunities to travel outside Nigeria to attend international workshops, which has widened access to world class education and training, resulting in improved delivery of quality cancer care in Nigeria Increased global visibility of National Hospital Abuja through representing Nigeria in the 63rd International Atomic Energy Agency general assembly in September 2019 in Vienna, Austria, and an invitation to represent Nigeria at the UN Disarmament Conference in New York City in May 2020 (postponed because of COVID-19) Increased respect and enhanced image of the National Hospital Abuja within the scientific community Improved the quality of academic lectures to resident doctors and undergraduate medical students resulting in the fostering of new mentoring relationships within Nigerian hospitals and academic medical centers

Abbreviations: COVID-19, coronavirus disease 2019; HIC, high-income country; ICEC, International Cancer Expert Corps; LMICs, low- and middle-income countries; NGO, nongovernmental organization.

BOX Perspectives From a Mentee and Mentor on Their Mentorship

Mentee: Taofeeq Abdallah, IGE, PhD, Chief Consultant Physicist and Head of Medical Physics Department, National Hospital, Abuja, Nigeria

Mentor: Manjit Dosanjh, PhD, Senior Advisor for Medical Applications, European Organization for Nuclear Research (CERN), Geneva, Switzerland

**Taofeeq Abdallah:** The relationship between Manjit and myself since our first meeting in CERN in 2017 has been more than awesome. She brought a fresh perspectives between a mentor and mentee by trying to identify real-time with the situation in the LMIC’s and this has propelled me and my colleagues on this “side of the divide” to push ahead even more vociferously knowing fully well that we can always rally for support anytime that this is needed and she has never disappointed in all the occasions – always rising up to the challenge and offering advice that are most accurate and incisive.

The tangible benefits that this international mentoring relationship have engendered has been first to our numerous patients who have in one way or the other benefited from very rich advice that Manjit has been able to offer from time to time – raising our spirits even in the face of arduous and unfavorable conditions. Since the relationship impacts our patients, this has equally been of great benefit to me professionally and has had a concomitant net benefits to my hospital and even my interactions with colleagues in the region as the president of our professional association (FAMPO – Federation of African Medical Physics Organizations).

**Manjit Dosanjh:** I got to work with and to know Taofeeq much more closely when the Science and Technology Facilities Council (STFC) team started to prepare a proposal to conduct an Accelerator Design Study (ADS) for a medical linear accelerator (LINAC) for Overseas Development Agency countries to be submitted to the Global Challenges Research Fund.

At my suggestion, both Taofeeq and Simeon Chinedu Aruah were invited to participate in the preparation of the ADS to advise the STFC team about both clinical and medical physics challenges of LINAC use in Nigeria. During the period of the development of the ADS proposal, I realized that Taofeeq and Simeon were not used to communicating and working closely with each other. This fact provided a great opportunity for me to help bridge that gap and build a closer working relationship between them.

Since then, I have been guiding Taofeeq in how to prepare and submit his own projects; he led the last one with myself as a co-applicant. We are now working on a questionnaire gathering information for optimizing a LINAC prototype for future machines suitable for challenging environments. Also, David Pistenmaa and I accepted Simeon and Taofeeq’s invitation to contribute to peer-reviewed manuscripts that they originated and enjoyed the camaraderie in doing so. What has been most rewarding to us over the last 2 years has been to see not only Taofeeq become a more understanding and caring leader but also to see the relationships between him and Simeon and their departments growing. These improving collaborations will continue to enhance the quality of treatment of patients with cancer and the reputation of National Hospital Abuja.

**Figure:**
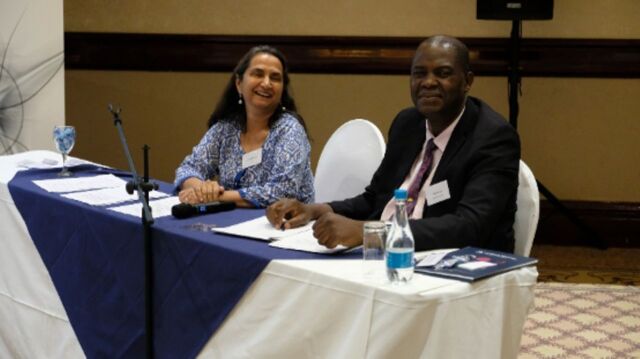
Manjit Dosanjh with Taofeeq Ige co-chairing a session at a workshop on radiation therapy treatment systems held in Gaborone, Botswana. © 2019 Manjit Dosanjh

Dr. Onyinye Balogun, a radiation oncologist from Weill Cornell Medical School, has established training programs in Armenia that have enabled the radiation oncologists to jump forward a few decades in radiation oncology from 2-dimensional radiation therapy to 3-dimensional techniques. Training and ongoing telemedicine case discussions enable further advancement in techniques that are less toxic and, by allowing higher doses, more effective. Her work and that of her mentors led the dean to establish a global oncology initiative at the medical school.

Surbhi Grover, MD, completed an MPH degree under mentorship advice and with support from the University of Pennsylvania. She has been hands-on in Botswana establishing evidence-based cancer care guidelines. This work is a major advance in care and has transformed the strategies to manage stock for chemotherapy as part of comprehensive care plans. As one of the first radiation oncologists to be on the ground in global health, her program is a highly sought-after rotation for residents interested in pursuing careers in global health.

**Figure:**
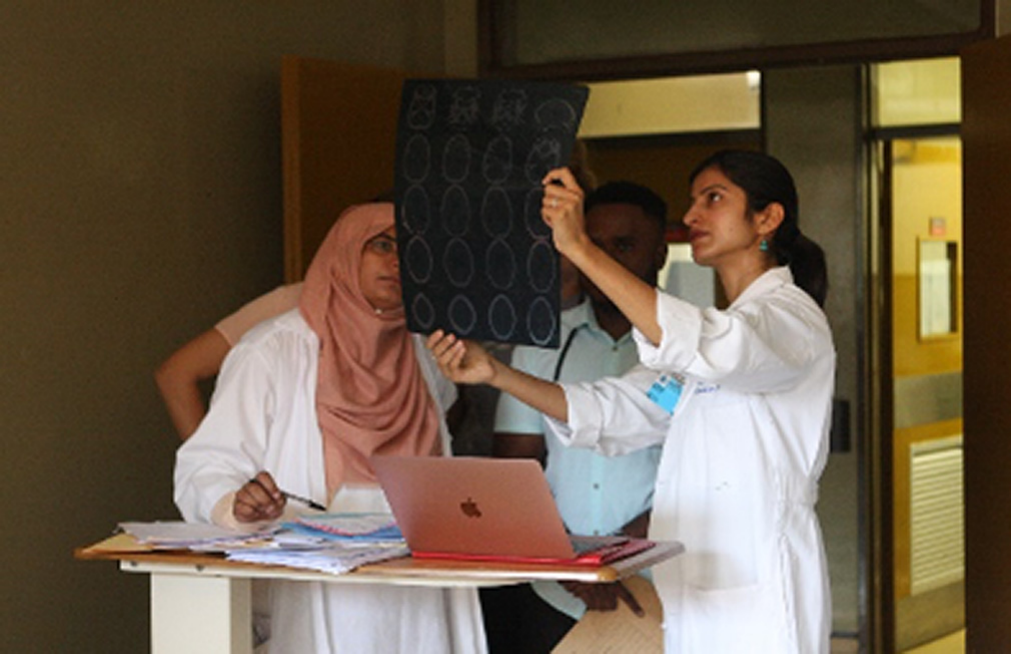
Dr. Surbhi Grover teaches staff in Botswana on the details of a radiation therapy field. Photo credit: ©2015 Surbhi Grover

Kristin Schroeder is a pediatric hematologist-oncologist from Duke University who has helped establish pediatric cancer care in Tanzania. She has not only implemented a comprehensive cancer care infrastructure, but also helped establish a nongovernmental organization to provide care to any child with cancer.

Taofeeq Abdallah is a medical physicist in Nigeria who has established, under the mentorship of CERN, education and training networking to enhance safety and teach a sophisticated technique for radiation therapy.

**Figure:**
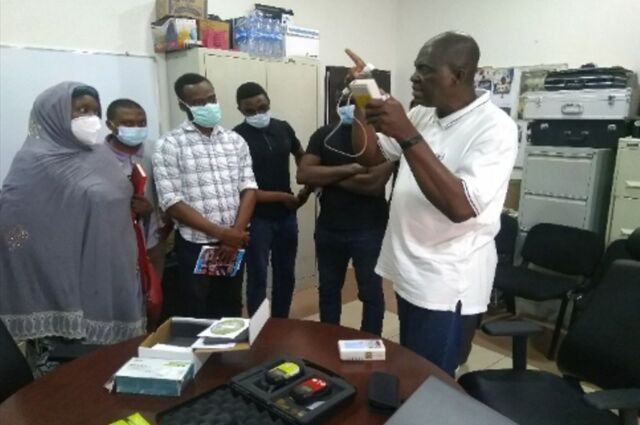
Taofeeq Ige, a medical physicist, educates staff on the technical specifications for radiation dose measurement in Abuja, Nigeria. Photo credit: ©2017 Taofeeq Ige

Simeon Aruah, MD, MPH, is a young lecturer in radiation and clinical oncology (includes medical oncology) in Nigeria, who has had a rapid growth in his academic career with assistance in conducting studies and preparing manuscripts and presentations from his mentors. His linkage with world-renowned academic mentors increased the visibility of his program and cancer care in Nigeria. His talent, enthusiasm, and confidence have grown, and he has already represented Nigeria at the International Atomic Energy Agency general assembly and will do so at the UN Disarmament Conference in New York City.

Interest in careers involving global oncology has surged with these pioneering examples, including program leaders willing to support trainees and faculty as part of a career path. The experience of the co-authors of this article can attest to the positive impact that the mentor-mentee relationship has on stimulating transgenerational idea sharing and generating energy and a positive outlook for what can be done, despite challenges that appear discouraging.

## OPPORTUNITY FOR A BROAD RANGE OF EXPERT MENTORS

Improved health care in general, building on the spectrum of cancer care from prevention through diagnosis, treatment, and long-term follow-up care, are the deliverables. Enabling this goal requires contributions including and *well beyond* patient care delivery from a broad range of experts, as shown in Table 2.

**TABLE 2.** Broad Spectrum of Expertise Needed for Complex System Solutions in Cancer Care

**Medical**	**Science, Non-MD**	**Support**
Radiation, medical, and pediatric oncologists Palliative care Surgeons including subspecialists Nurses Pathologists Radiologists General internists Primary care Infectious diseases Gerontologists Pharmacologists Psychologists Public health Emergency medicine	Prevention and screening Epidemiologists Medical physicists Technologists Basic and translational scientists Medical education Treatment guidelines Statisticians Social scientists Political scientists Regulatory affairs specialists Pharmacists Data management and big data science	Educational tools Finance Hospital/medical administration International policy Patient advocacy Economists Sociologists Social workers Cultural experts Diplomats Communications Cancer survivors Information technology Legal Development

Critically, the focus on cancer as part of the health care system encompasses the other NCDs—respiratory, cardiovascular, and metabolic diseases—and infectious diseases that are linked to both cancer etiology and complications of treatment. Cancer is a logical focus in that it has a sense of urgency, similar to infectious diseases, and it can be a focal point for community involvement. Thus, an opportunity exists for volunteers with a wide range of skills and expertise, including medicine, a broad range of scientific disciplines, and other professions (“broad support” such as cultural experts, communications, logistics, finance, and legal), to effectively transfer knowledge and wisdom, while reducing the expense associated with personnel. Thus, global mentorship teams can educate one another and provide mentorship to the local champions who are building programs in underserved communities, enabling the geometric expansion of health care necessary to address the enlarging workforce shortfall. Cultural competency^1^ is essential, and it benefits from those with in-country experience. Answering the questions of “What can I do, and how do I do it?” is facilitated by structure with achievable expectations. For a mentor, the expectation is only a ∼20% time commitment (8 hours per week on average), with the vast majority of the mentoring by planned teleconferencing (with some bidirectional travel possible) through protocol- and guideline-based care, rather than individual case management. As described by Crisp,^22^ knowledge and models for care will also evolve from mentees to mentors through reverse innovation.

Cancer is a logical focus in that it has a sense of urgency, similar to infectious diseases, and it can be a focal point for community involvement.

## DISCUSSION

In our opinion, raising the mandatory retirement age or eliminating it altogether—as is happening in many societies—presents new opportunities for those affected by necessary transitions in leadership in health care organizations, governmental and international diplomatic organizations, and academia that free senior personnel to mentor within step-down roles at work or later in retirement. This transition of lives and careers provides exceptional opportunities for the older generation to pass on its knowledge and wisdom to the younger generations through mentorship, while enhancing the quality of their own lives in their later years. This sentiment was recently highlighted by Jane E. Brody in *The New York Times* “Want to leave a legacy? Be a mentor.”^23^

Retirement provides exceptional opportunities for older individuals to pass on their knowledge and wisdom, while enhancing their own quality of life.

For those interested in a continued purpose in life related to their profession, addressing both healthspan and lifespan^4^ ^,^ ^24^ requires opportunities to use their skills by volunteering time and expertise. This trend (“purposeful healthspan”) utilizes this experience at a low cost for a wide range of organizations and interest groups, such as professional societies, religious organizations, and specific social causes. The model presented here is based upon periodic short visits followed by sustainable commitments and continuous mentoring of those working on site through teleconferencing. The model creates long-standing relationships and programs built around the broad range of skills transferred to the mentored specialists and staff in the local communities (Table 2).

Several similar large-scale mentorship model programs have found the keys for successful implementation to be convenience, flexibility, and purposefulness. Such examples include the following: (1) The AARP Foundation Experience Corps’ intergenerational volunteer-based tutoring program designed to help elementary school students improve their reading levels and to help older adults enrich their lives through literacy,^25^ (2) the Returned Peace Corps Volunteers,^9^ (3) the Japanese government’s “The Community-based Integrated Care System” providing comprehensive up-to-the-end-of-life support in every community,^26^ (4) Singapore’s Action Plan for Successful Aging that enables seniors to learn new skills in joyful endeavors and to deploy these skills, and (5) the National University Health System and the National University of Singapore’s work with multiple government agencies to enable whole precincts to exploit the elements of successful aging, thus future-proofing Singapore as a livable city for people of all ages.^27^ This “whole of society” and “whole of government” approach will enable societal change to take place and remain sustainable.

## CONCLUSION

The confluence of opportunities for continuity and the spectrum of expertise from senior mentors to those early in their career has not been more apparent than in the current COVID-19 pandemic. On the one hand, the call for retirees to return to health care^28^ speaks to how senior and world-renowned experts’ skills are useful for their primary expertise and for their role modeling, gravitas, and potential direct support. Yet, on the other hand, greater awareness of such usefulness and the presence of senior experience and wisdom might have averted the wholesale dismissal of 7,300 Peace Corps volunteers^29^ and fostered a more appropriate transition.

Older individuals have opportunities to serve society and humanity. Such opportunities (1) provide a career capstone, (2) allow timely transfer of institutional responsibility to next-generation leaders, (3) establish mentorship relationships for world-renowned experts with dedicated professionals in underserved and geographically remote health care regions, (4) provide expensive expertise at “volunteer prices,”^11^ ^,^ ^20^ (5) present a model for geometric expansion of diverse expertise and innovative technology that enables development of the capacity to effectively address the burgeoning burden of cancer and other NCDs, (6) establish a mentor-based career path for altruistic human service that is an endangered species in the current “bottom line” finance-driven health care system, (7) emphasize the importance of cultural competence and listening, and (8) utilize a systems solution approach to improve health care in LMICs by developing and sustaining local champions. The presence of a gap that can be filled in a rather short timeline from mentor to mentee to LMIC mentee speaks to the need and impact.

Whether being engaged in purposeful activities, such as those described in this article, will increase the length of one’s lifespan is under study. Such study includes understanding the impact of aging on the workplace.^30^ Interestingly, coinciding with this current article, Dzau et al.^31^ recently announced “The National Academy of Medicine Grand Challenge in Healthy Longevity.” What is undeniable is that the benefit of such activities can meaningfully increase the breadth of one’s experiences and contributions to society. Serving as a senior mentor to mentees in resource-poor regions of the world can have a spectacular impact on the goal of rectifying the staggering lack of access to care for patients with cancer and other NCDs in those regions. In addition, by improving the infrastructure of cancer care facilities and expanding the breadth of expertise available to them, these facilities can serve as focal points for the development of sustainable on-the-ground programs that can have substantial health and economic benefits beyond cancer care. Transformational models, as outlined in this article, offer opportunities for visionary investments, altruistic contributions, and exciting and meaningful action for a purposeful aging and improved healthspan.

## Supplementary Material

20-00108-Coleman-Supplement.pdf

## Notes

**Peer Reviewed**

**First published online:** October 15, 2020
