# Supplementary material for: Capturing Acquired Wisdom, Enabling Healthful Aging, and Building Multinational Partnerships Through Senior Global Health Mentorship
Source: Glob Health Sci Pract. 2020 Dec 23;8(4):626–37. doi: 10.9745/GHSP-D-20-00108 (PMC7784062; doi:10.9745/GHSP-D-20-00108)
Supplement: 20-00108-Coleman-Supplement.pdf [file 20-00108-Coleman-Supplement.pdf]

Supplement to: Coleman CN, Wong JE, Wendling E, et al. Capturing acquired wisdom, enabling healthful aging, and building multinational partnerships through senior global health mentorship. *Glob Health Sci Pract*. 2020;8(4). https://doi.org/10.9745/GHSP-D-20-00108

Supplement

I. Tapping into senior expertise and transgenerational mentorship.

Problems as large as the gap in global healthcare may seem to be "too hard" to address but specific examples can make the solution less daunting and even an exciting personal challenge. Six examples of mentorship involving co-authors of this paper are presented to demonstrate the power of mentorship and trusted relationships in creating new paradigms and successful partnerships.

A. Societal mission for academic medicine.

The creation of a global health initiative by a chain of mentors committed to healthcare, addressing societal inequality, patient empowerment and willingness to take on problems is often considered "too hard". That it can be done in academic oncology was shown by Dr. Henry and Leah Kaplan who were world-renowned role models at Stanford to Dr. David Pistenmaa (former Chair of the Department of Radiation Oncology at the University of Texas Southwestern Medical Center) and Dr. Norman Coleman (former Chair of Harvard Medical School Joint Center for Radiation Therapy and Chief of the United States (US) National Cancer Institute's (NCI) Radiation Oncology Branch). At a 1994 panel "Cancer at the Crossroads: A report to Congress for the Nation"[1]], Coleman's chance meeting with Ellen Stovall, then President and CEO of the National Coalition for Cancer Survivorship (NCCS)[2] inspired Coleman to become more involved in community outreach to underserved communities.[3] Stovall encouraged Nina Wendling, recently retired Chief Operating Officer of NCCS, to join ICEC Operations. In addition to Pistenmaa upon his return to Washington DC., other Coleman colleagues, many going back decades (including co-authors Dr. Harmar Brereton, Donna O'Brien, and Dr. Mary Gospodarowicz), accepted leadership positions on ICEC Boards and programs. Accomplishments and vision are summarized in "A Broad Impact for Global Oncology" (Coleman, Wendling and Pistenmaa 2019) and are exemplified by the "Young Investigators' Conference: Pioneering Action for Global Cancer Care".[4] Mentorship starting from the Kaplans is now in its 4th generation.

B. Understanding technology's potential reach to underserved humanity.

The joint biannual integrated meetings of the International Conference on Translational Research in Radio-Oncology (ICTR) and Physics for Health in Europe (PHE) organized by Professors Jacques Bernier and Manjit Dosanjh, a radiation biologist from CERN,[5] brings clinicians and medical physicists together. Following a presentation by Coleman on Global Health at their 2014 meeting Dr. Ugo Amaldi, a creative pioneering leader in high energy physics, stated "we can build a better linear accelerator for the developing world". Amaldi's offering his knowledge, expertise and passion to invent novel technology for global cancer care was championed by his colleagues and trainees resulting in a series of workshops involving physicists and engineers

from CERN, the UK Science and Technology Financing Council and others, as highlighted in the recent ENLIGHT Network[6] newsletter.[7] This example of cross fertilization of innovative technological expertise, clinical need and commitment to social justice that is being adapted by early career global health leaders represents a 3rd generation transfer of creative knowledge starting with Amaldi.

C. Global cancer care - committed, tireless leadership by example.
Perhaps based on inspiration from working with Kaplan during her training at Stanford when she met Coleman and Pistenmaa, Gospodarowicz has been and remains a transformational figure in Canadian radiation oncology and a pioneer in global health. Leading by example when she was President of the UICC [8], she assembled a broad-based team of world experts including Rifat Atun, professor of global health specializing in health systems, Dr. David Jaffray , medical physicist and Dr. Danielle Rodin, then a radiation oncology resident, that prepared the in-depth GTFRCC report noted above and demonstrated that curing cancer is cost effective for a poor country. Gospodarowicz pioneered a career path in global oncology for trainees, junior faculty and now a 3rd generation of medical students best exemplified by her former trainee, Rodin who is forging a global oncology career path with her early-career colleagues[9] and is bringing expertise in economics and outcomes analysis to global health.[10]

D. Trusted decades-long relationships build unique partnerships.
As noted by Margaret Meade, small groups of dedicated determined people are a major force in changing the world.[11] These efforts, whether involving smaller or major organizations and institutions, invariably involve trusted relationships. ICEC began with international professional relationships that included Professors Ed Liu, Norman Coleman, Michael Friedman, John Wong, and Soo Khee Chee of the now completed Singapore Cancer Syndicate. Wong and Chee are founders of ICEC Hubs in Singapore, a nation that is a driving force in addressing aging, an issue recognized as critical to its future.

Trusted professional relationships also have led to the pioneering inclusion of geographically isolated populations, often indigenous populations[12] in UICs, in global health because their healthcare situation may be quite similar to that in LMICs. This is illustrated by the success story of growing collaborations between Canada and their First Nations programs with the American Indians, specifically the Lakota Sioux American Indians centered in Rapid City South Dakota.[13] The Walking Forward innovative program was started by the U.S. National Cancer Institute's (NCI) Cancer Disparities Research Partnership Program (CDRP) with Dr Daniel Petereit, a community radiation oncologist as the Principal Investigator.[13] This concept made the local team the grant recipient rather than channeling funds through an academic center and secondarily to the local community thereby empowering the local champions. Mentorship from

experts at the NCI to the grantees in the underserved community has reached a 3rd generation successfully.

E. Engaging community practitioners.

Building in part from the success of the above CDRP program, the NCI's pioneering Community Cancer Centers Program (NCCCP)[14] of which ICEC Board member Donna O'Brien was a leader, targeted community practitioners who see 85% of patients with cancer in the US. The NCCCP program model marshaled the expertise of community physicians both to work with and to learn from community hospital cancer programs in 22 states and to support a learning collaborative for benchmarking performance. The result was improved evidence-based care and increased access to clinical trials, particularly for underserved populations.[15,16]

Community oncologists also are becoming concept and program leaders in global health. One example is Chartrounds pioneered by Dr. Patricia Hardenberg who had trained at Harvard with Coleman. Chartrounds is fulfilling a need for ongoing expertise in patient care by facilitating disease management teleconferences worldwide. Private community practice-based Presidents of the American Society of Radiation Oncology (Drs. Tim Williams and Thomas Eichler) have contributed their knowledge and experience in major leadership roles by making global health an integral part of practice and by mentoring young practitioners as they engage in this new career opportunity, 2nd generation mentorship.

F. Mentorship and twinning programs - academia begins to embrace global health careers.

Dr. Augustine M.K. Choi, Dean of Weill Cornell School of Medicine, has implemented a comprehensive institution-wide mentoring program at Cornell – "everyone mentors, everyone is mentored". Dr. Silvia Formenti, ICEC Board member and Chair of Cornell's Department of Radiation Oncology, in the interest of helping the Weill Cornell Department of Global Medicine expand its program of global cancer initiatives, recognized that Dr. Onyinye Balogun, a newly recruited radiation oncologist whose family was from Nigeria and is pioneering a career path in global oncology, could provide that assistance. To provide mentoring for Balogun in the practical, business side of a practice, Formenti invited Dr. Harmar Brereton, a retired community medical and radiation oncologist with experience running a complex, multi-location comprehensive cancer care practice to mentor Balogun (at no cost). Formenti's foresight and the Dean's support represent a successful collaboration to support the medical school's missions of mentoring, expanding its global health effort and formalizing a new career path. This is a nascent 3rd generation mentorship. In this web of trusted partnerships, Coleman invited Brereton, with whom he has a 40-year friendship, to join the ICEC Board.

G. Peace Corps and diplomacy last a lifetime: The ripples grow outward.

The expansion of mentorships between individuals not necessarily within the same institution is a dynamic attribute of the ICEC structure. It also became apparent that it may be necessary for even a senior Mentor to need advice and counsel on aspects of their assignment. While often well-travelled not every oncologist has lived in Lesotho, Botswana or Bangladesh. And even a one week visit that would help in establishing an interpersonal relationship might not necessarily result in a comprehensive understanding of the local political and physical realities of a distant LMIC location. Thus, arises the potential use/assignment to the mentor of what could be designated as a Cultural Mentor. At this writing work is being done by Larry Roth, a Peace Corps volunteer in Lesotho (Lesotho 1. 1967-69) to build a linkage to network of Returned Peace Corps Volunteers [10] and the cadre of retired senior staff; Country Directors, Public Health Country Directors, (Public Health being a primary Peace Corps focus). These individuals would provide key insights into the on-the-ground realities, not limited to infrastructure, government support, and the people's view of 'modern health practices' and providers. Providing another productive use of an individual's well-earned wisdom to make one specific location a slightly better place and a stepping-stone to lifelong mentor-mentee relationship.

II. Mentee narratives, "in their own words."

A. Weill Cornell Medicine, New York, USA and National Center of Oncology, Yerevan, Armenia
Mentee: Onyinye Balogun, MD
The initial program between Weill Cornell and the National Center of Oncology, Yerevan, Armenia, was to provide an educational and ongoing training program to guide the transition from using conventional 2D to 3D conformal treatment planning for the treatment of cancer with radiation therapy, especially for breast cancer. In Armenia, 62% of breast cancer cases present in stage 3 or 4 and where Armenia has the 4th highest mortality rate from breast cancer in the world. The most recent project concerns the proper implementation of image-guided brachytherapy for cervical cancer. These two problems were addressed in three visits to the cancer center in Yerevan with didactic lectures to staff and hands-on training for the implementation of the above services. Dr. Balogun continues communication with the staff she helped train through teleconferences focused on patient discussion and peer review.

Through these efforts, Dr. Balogun initiated the global oncology initiative at Weill Cornell Medicine and established one of the first "Twinning" programs that link emerging cancer treatment programs in an LMIC with an advanced cancer treatment program represented by the International Cancer Expert Corps (ICEC).

Dr. Balogun benefited from Dr. Silvia Formenti's and Dr. Harmar Brereton's mentoring in multiple ways including, 1) ensuring protected time and financial support for the development of

a career in global oncology when no clear path existed; 2), offering guidance and training in the critical areas of decision making and time management, 3) permitting the opportunity to work "in-country" in Yerevan for a 6-week program, and 4) providing for the recruitment of an additional physician on behalf of the ICEC who will continue and expand the relationship with the National Center of Oncology in Yerevan. This now well-established program has improved the National Center of Oncology's capacity to treat all cancers, and especially breast cancer, with 3D treatment planning and will lead to better outcomes, increase the participation of additional physicians in the radiation oncology service and will foster ongoing and future on-site and remote communications and education. Most importantly, the program has fostered a strong mutual understanding and respect among all physicians and health care professionals involved - an invaluable and important unintended benefit welcome by all.

Mentor: Silvia Formenti, MD
Chairman
Department of Radiation Oncology, Weill Cornell
Associate Director of the Meyer Cancer Center and Radiation Oncologist in Chief,
New York Presbyterian Hospital

Since meeting Dr. Balogun "Onyinye" as a resident when we both were working at NYU, I was impressed by her professionalism and dedication to our field. In addition, Onyinye is a natural leader. We were fortunate to be able to recruit her to Weill Cornell when I moved from NYU and was ecstatic to learn about her interest in global health, with a focus on radiation oncology. Since then, my relationship with Dr. Balogun has evolved, whereby while I remain her Mentor and enthusiastic supporter, I continue to learn from her and her experience in the field.

My job also includes giving some direction to the academic translation of her ideas and experiences, and several publications have resulted from this process. This component includes work within the Institution to map a global oncology career pathway that recognizes the accomplishments in this new area and in enabling philanthropic support in this direction.

In summary, I remain committed to assuring unconditional support to Dr. Balogun's internationally focused career in radiation oncology.

Mentor: Harmar Brereton, MD
Clinical Professor of Medicine
Geisinger Commonwealth School of Medicine and Clinical Assistant Professor of
Radiation Oncology, Weill Cornell

Supplement to: Coleman CN, Wong JE, Wendling E, et al. Capturing acquired wisdom, enabling healthful aging, and building multinational partnerships through senior global health mentorship. *Glob Health Sci Pract*. 2020;8(4). https://doi.org/10.9745/GHSP-D-20-00108

Dr. Brereton's interest in healthcare in LMIC began when he spent time practicing in the American Virgin Islands taking care of indigent patients. His background, which includes training in internal medicine, hematology, medical oncology and radiation oncology, the establishment of cancer treatment and research programs came together with the opportunity offered by Dr. Silvia Formenti at Weill Cornell to mentor in the development of a global oncology program. Dr. Brereton's ongoing, consistent advice, guidance and collegial respect serves to help Dr. Balogun in her efforts to transform global cancer care.

B. Princess Marina Hospital, Gaborone, Botswana and University of Pennsylvania, Philadelphia, Pennsylvania, USA
Mentee: Surbhi Grover, MD, MPH
Assistant Professor of Radiation Oncology
Perelman School of Medicine
University of Pennsylvania
Princess Marina Hospital, Gaborone, Botswana

Dr. Grover's interest in public health developed during college in NYC at Columbia University and medical school at Harvard. Today, she serves as an Assistant Professor of Radiation Oncology at the University of Pennsylvania (US) and Adjunct Senior Lecturer at the University of Botswana, focusing on public health endeavors and cost-effective clinical initiatives to improve access to cancer care and outcomes of care in developing countries. Key mentors played and continue to play an essential role in Dr. Grover's career, supporting and guiding her interest to improve access to radiation oncology globally.

Dr. Grover's current efforts in Botswana center around four main pillars: clinical care at Princess Marina Hospital; research in the framework of a collaboration of UPENN department of radiation oncology with the University of Botswana; education through U54 (a mentoring core collaborative grant between UPenn and UB grant aimed to develop research capacity in HIV and cervical cancer among junior faculty at UB); and technical assistance to Ministry of Health (MOH) to develop cancer guidelines for top 10 cancers in Botswana.

Significant mentors to Dr. Grover include Stephen Hahn, MD, FDA Commissioner, Former Chair, Department of Radiation Oncology Perelman School of Medicine, University of Pennsylvania; James Metz, MD, Chair, Department of Radiation Oncology, Perelman School of Medicine, University of Pennsylvania; Edward L. Trimble, MD, MPH, Senior Advisor for HPV and Global Cervical Cancer Control, Office of the Director, National Cancer Institute; and C. Norman Coleman, MD, Radiation Oncology Branch, Head, Experimental Therapeutics Section, Associate Director, Radiation Research Program, DCTD, NCI, Senior Medical Advisor, Office of the Assistant Secretary for Preparedness and Response, DHHS.

Supplement to: Coleman CN, Wong JE, Wendling E, et al. Capturing acquired wisdom, enabling healthful aging, and building multinational partnerships through senior global health mentorship. *Glob Health Sci Pract*. 2020;8(4). https://doi.org/10.9745/GHSP-D-20-00108

Mentor: Stephen Hahn, MD
FDA Commissioner
Former Chair, Department of Radiation Oncology Perelman School of Medicine, University of Pennsylvania

Mentor: James Metz, MD
Chair, Department of Radiation Oncology, Perelman School of Medicine, University of Pennsylvania

Drs. Hahn and Metz helped create and promote global health as an academic career path, by supporting Dr. Grover's faculty rotation in Botswana including salary support as a faculty member at UPenn. Given the status of UPenn in academic radiation oncology this action spoke volumes and is one of the commitments that have empowered other departments to follow.

C. Bugando Cancer Center, Mwanza, Tanzania And Duke Children's Hospital & Health Center, Durham, NC, USA
Mentee: Kristin Schroeder, MD, MPH
Pediatric Hematology-Oncology Specialist, Pediatric Neuro-oncologist
Bugando Cancer Center in Mwanza, Tanzania
Duke Children's Hospital & Health Center, North Carolina, USA

Dr. Kristin Schroeder's efforts in pediatric oncology in Mwanza, Tanzania, have been fostered by a key mentoring relationship between Dr. Schroeder and Nelson Chao, MD, Director, Global Cancer at the Duke Global Health Institute. Together, they leveraged each other's interest in global health and developed the pediatric cancer care program at the Bugando Cancer Center in Mwanza, Tanzania.

With the thoughtful advice and guidance of Chao mand others, Dr. Schroeder and the team of healthcare providers at Bugando Cancer Center continue to improve the delivery of pediatric cancer care and patient outcomes. Dr. Schroeder recognizes the importance of her relationship with Chao and has herself initiated mentoring relationships with nearly 20 individuals, including Fulbright scholars, masters level students, oncology fellows, nurses, residents, medical students and undergraduate students.

The pediatric cancer program at the Bugando Cancer Center has, too, benefitted from the partnership, including the establishment of a patient navigator program, the development of a pediatric cancer clinical database, and the establishment of a hospital-based cancer registry. Additionally, streamlining access to treatment and the implementation of care protocols and

research programs related to specific cancers have improved outcomes. To ensure access to care for pediatric patients, Schroeder, encouraged by Chao, founded an NGO, the International Cancer Care and Research Excellence Foundation (iCCARE). The foundation covers treatment costs for BMC pediatric cancer patients, provides other resources and support, including a hostel where patients and families can stay while receiving treatment and provides social work and child life services to pediatric cancer patients.

To date, the survival rate at BMC has increased from 19 percent to 44 percent, treatment abandonment rate has decreased from 47 percent to 20 percent, and community cancer awareness and education programs have been developed. Schroeder continues her work as a Pediatric Hematology-Oncology Specialist, Pediatric Neuro-oncologist at both Bugando Cancer Center in Mwanza, Tanzania, and the Duke Children's Hospital & Health Center, in Durham, NC, USA.

Mentor: Nelson Chao, MD, MBA
Donald D. and Elizabeth G. Cooke Professor
Chief, Division of Hematologic Malignancies and Cellular Therapy/BMT
Director, Global Cancer, Duke University School of Medicine

Time to give back.
"We make a living by what we get, we make a life by what we give." — *Winston Churchill*

Growing up in Brazil and observing the tremendous income and health care inequities left a major impact on Nelson Chao. After oncology training at Stanford University with Norm Coleman, he stayed on as faculty at Stanford in oncology (stem cell transplant). In 1996, he moved to be the chief of stem cell transplant at Duke University. In 2013 he was tasked by the Duke Global Health Institute and the Duke Cancer Institute to develop a global oncology program. With the goal of first providing care, he hired Kristin Schroeder, a Duke trained pediatric oncologist. Together they developed the pediatric cancer care program at the Bugando Cancer Center in Mwanza, Tanzania. She has had significant challenges but also great successes such as improving survival dramatically in these children through introduction of a navigator program, a hostel and standard protocols for care. In turn, Dr. Schroeder has now mentored 19 individuals including 2 Fulbright scholars, 5 masters level students, 1 oncology fellow, 2 nurses, 1 resident, 3 medical students and 3 undergraduate students. She has also help organize pediatric regimens across the country pulling together the three institutions where pediatric cancers are treated now with uniform protocols. The lessons learned in Tanzania are applicable to many LMIC settings and the ability to build research teams further the scientific efforts around the area of non-communicable diseases.

Supplement to: Coleman CN, Wong JE, Wendling E, et al. Capturing acquired wisdom, enabling healthful aging, and building multinational partnerships through senior global health mentorship. *Glob Health Sci Pract*. 2020;8(4). https://doi.org/10.9745/GHSP-D-20-00108

D. National Hospital, Abuja, Nigeria and the European Organization for Nuclear Research (CERN), Geneva, Switzerland

Mentee: Taofeeq Abdallah IGE, PhD
Chief Consultant Physicist and Head of Medical Physics Department
National Hospital, Abuja, Nigeria

The relationship between Manjit and myself since our first meeting in CERN in 2017 have been more than awesome. She brought a fresh perspectives between a mentor and mentee by trying to identify real-time with the situation in the LMIC's and this has propelled me and my colleagues on this "side of the divide" to push ahead even more vociferously knowing fully well that we can always rally for support anytime that this is needed and she has never disappointed in all the occasions – always rising up to the challenge and offering advise that are most accurate and incisive.

The tangible benefits that this international mentoring relationship have engendered has been first to our numerous patients who have in one way or the other benefited from very rich advice that Manjit have been able to offer from time to time – raising our spirits even in the face of arduous and unfavorable conditions. Since the relationship impact our patients, this has equally been of great benefit to me professionally and has had a concomitant net benefits to my hospital and even my interactions with colleagues in the region as the President of our professional association (FAMPO – Federation of African Medical Physics Organizations).

The sustainability of these unique relationship is predicated on passing on the knowledge and skills garnered to the next generation therefore deliberate attempts have been made and under gradual implementation to "pass on" the core elements of this mentor-mentee fraternity to my junior colleagues both in the hospital and at the national and regional professional platforms such that they are able to approach me at any time for varied advice and reasons. For example, I have been able to rally my post-graduate students and junior colleagues in the department to submit useful abstracts and seminal presentations during our annual scientific conferences and celebrations of the International Day of Medical Physics (IDMP) both in my country – Nigeria and in the African region. In recognition of this efforts, I got the IDMP Award 2017 for Africa by the IOMP (International Organization of Medical Physics) and this was presented to me at the World Congress in Prague (Czech Republic) in June 2018. The award was presented for promoting medical physics to a larger audience and highlighting the contributions medical physicists make for patient care.

Joint and mutually beneficial cross-border networking projects will be quite helpful in the immediate and foreseeable future. Such projects that will positively impact the prognosis and treatment outcomes of our numerous cancer patients and also help in disseminating up to date

knowledge and skills to my post-graduate students, trainees and junior faculty members both in the hospital and the university. These can be facilitated by short scientific visits abroad, WebEx meetings via videoconferencing and webinar series on a monthly or bi-monthly basis. Some of the ongoing efforts were recently published in the Medical Physics International journal (MPI Vol.7 No.3) and can be accessed via www.mpijournal.org /MPI-v07i03.aspx

Mentor: Manjit Dosanjh, PhD
Senior Advisor for Medical Applications, CERN

I met Taofeeq at the CERN- and ICEC-sponsored workshop at CERN in October 2017 that was dedicated to defining the design characteristics of a novel medical linear accelerator for challenging environments such as where he works in Abuja, Nigeria. We much appreciated his insightful contributions in that workshop and in two subsequent workshops sponsored by CERN, ICEC and STFC: in Stretton, UK in 2018 and in Gaborone, Botswana in 2019. I got to work with and to know Taofeeq much more closely when the STFC team started to prepare a proposal to conduct an Accelerator Design Study (ADS) for a medical linear accelerator for Overseas Development Agency (ODA) countries to be submitted to the GCRF (Global Challenges Research Fund). At my suggestion, both Taofeeq and Simeon Chinedu Aruah were invited to participate in the preparation of the ADS to advise the STFC team about both clinical and medical physics challenges of LINAC use in Nigeria. During the period of the development of the ADS proposal, I realized that Taofeeq and Simeon were not used to communicating and working closely with each other. This fact provided a great opportunity for me to help bridge that gap and build a closer working relationship between them. Since then, I have been guiding Taofeeq in how to prepare and submit his own projects; he led the last one with myself as a co-applicant. We are now working on a questionnaire gathering information for optimizing a LINAC prototype for future machines suitable for challenging environments. Also, David and I accepted Simeon and Taofeeq's invitation to contribute to peer-reviewed manuscripts that they originated and enjoyed the camaraderie in doing so. What has been most rewarding to us over the last two years has been to see not only Taofeeq become a more understanding and caring leader but also to see the relationships between him and Simeon and their departments growing. These improving collaborations will continue to enhance the quality of treatment of patients with cancer and the reputation of National Hospital Abuja.

E. National Hospital, Abuja, Nigeria and the International Cancer Expert Corps (ICEC)
Mentee: Simeon Chinedu Aruah (MD, MPH, FWACS)
Consultant Radiation and Clinical Oncology
National Hospital Abuja, Nigeria
Lecturer University of Abuja College of Medicine, Nigeria
Head of Department Radiation and Clinical Oncology

Supplement to: Coleman CN, Wong JE, Wendling E, et al. Capturing acquired wisdom, enabling healthful aging, and building multinational partnerships through senior global health mentorship. *Glob Health Sci Pract*. 2020;8(4). https://doi.org/10.9745/GHSP-D-20-00108

National Hospital Abuja Nigeria
I was fortunate to have been invited to participate in an international workshop sponsored by CERN, STFC and ICEC at CERN in October 2017 where I met Drs. Manjit Dosanjh and David Pistenmaa. Since that time, their mentoring has been very important to the development of my career as Consultant Radiation and Clinical Oncologist at National Hospital Abuja and lecturer in the University of Abuja College of Medicine. Among the benefits to me of their mentoring are 1) academic growth through publications with them in top scientific oncology journals, 2) presentation of quality papers in different fora with their guidance and support , 3) opportunities to travel outside my country to attend international workshops which have widened my horizon and improved the quality of cancer care in Nigeria, 4) invitations to represent Nigeria in the 63rd International Atomic Energy Agency (IAEA) general assembly in September, 2019 in Vienna, Austria and at the UN Disarmament Conference in NYC in May 2020, both of which have increased global visibility of National Hospital Abuja where I work, 5) increased respect among my colleagues and an enhanced image of the National Hospital Abuja within the scientific community and 6) my overseas travel and international collaborations have had a positive impact on the quality of my academic lectures to our resident doctors and undergraduate medical students and, in turn, greater respect for me from them.
I attribute in large part my present status as Head of Department of Radiation and Clinical Oncology, National Hospital Abuja to the encouragement and support of and knowledge gained from my international mentors. To strengthen the relationship with my overseas mentors, it would be nice to expand on our current interactions through emails and phone calls by way of video conferencing or WhatsApp messaging. Such increased interactions could make additional useful contributions to my academic growth, clinical competence, career development and care of cancer patients.
Mentor: David A. Pistenmaa, MD, PHD, FACR
Chief Scientific Program Director
International Cancer Expert Corps

Mentor: Manjit Dosanjh, PhD
Senior Advisor for Medical Applications, CERN

It has been a privilege for us to work with Simeon since we met him at the CERN- and ICEC-sponsored workshop at CERN in October 2017 that was dedicated to defining the design characteristics of a novel medical linear accelerator for challenging environments such as where he works in Abuja, Nigeria. We greatly appreciated his enthusiastic contributions in that workshop and in two subsequent workshops sponsored by CERN, ICEC and STFC: in Stretton, UK in 2018 and in Gaborone, Botswana in 2019. We accepted his invitation to contribute to manuscripts that he has originated and enjoyed the camaraderie in doing so. Manjit also has communicated with Simeon regularly about various aspects both at professional and personal

levels, mentoring him about the importance of working together and collaborating with colleagues at his hospital and externally. Simeon has demonstrated awareness of his leadership role in setting an example for his staff to follow and how critical it is to have good relationships with other hospital departments to both deliver excellent treatment to patients and to create an effective working environment. What has been most rewarding to us has been to see Simeon be asked and accept greater responsibilities in developing the cancer research and treatment program at National Hospital Abuja (NHA) as Head of the Department of Radiation and Clinical Oncology. Simeon's participating in international workshops, being co-author of several manuscripts that have been published in peer-reviewed journals and invited to a number of important political and social events in Nigeria, Africa and internationally, has enhanced his professional standing in Nigeria and Sub-Sahara Africa (SSA). In this brief period, Simeon has been appointed to regional and national committees, represented Nigeria at a meeting of the International Atomic Energy Agency (IAEA) in Vienna in 2019 and was to have represented his country at a meeting at the UN in New York City in May 2020, since postponed because of the coronavirus. We look forward to watching his career evolve further as he works with others at NHA in the years to come.

REFERENCES

1. United States. National Cancer Advisory Board. Subcommittee to Evaluate the National Cancer Program. *Cancer at the Crossroads. A Report to Congress for the Nation.* National Cancer Advisory Board; 1994. Accessed March 1, 2020. https://books.google.com/books/about/Cancer_at_a_Crossroads.html?id=gJcIAAAAIAAJ

2. National Coalition for Cancer Survivorship. Home page. Accessed March 1, 2020. https://www.canceradvocacy.org

3. Wong RS, Vikram B, Govern FS, et al. National Cancer Institute's Cancer Disparities Research Partnership Program: experience and lessons learned. *Front Oncol.* 2014;4:303. CrossRef.

4. International Cancer Expert Corps. Young Investigators' Conference: Pioneering Action for Global Cancer Care. Accessed July 28, 2019. https://www.iceccancer.org/registration-opens-young-investigators-conference-pioneering-action-global-cancer-care/

5. International Conference on Translational Research in Radio-Oncology, Physics for Health in Europe (2016 conference). Accessed March 1, 2020. https://www.thegreenjournal.com/issue/S0167-8140(15)X0016-8?page=1

6. Dosanjh M, Amaldi U, Mayer R, Poetter R. ENLIGHT: European Network for Light ion hadron therapy. *Radiother Oncol*. 2018;128(1):76-82. CrossRef. PMID: 29625810

7. ENLIGHT newsletter. Radiation therapy treatment system for challenging environments. https://www.iceccancer.org/wp-content/uploads/2019/12/Enlight-Highlights-June-2019.pdf

8. Union for International Cancer Control. Home page. Accessed March 1, 2020. https://www.uicc.org

9. Rodin D, Yap ML, Grover S, Longo JM, et al. Global health in radiation oncology: the emergence of a new career pathway. *Semin Radiat Oncol.* 2017;27(2):118-123. CrossRef. PMID: 28325237

10. Rodin D, Burger EA, Atun R, et al. Scale-up of radiotherapy for cervical cancer in the era of human papillomavirus vaccination in low-income and middle-income countries: a model-based analysis of need and economic impact. *Lancet Oncol*. 2019;20(7):915-923. CrossRef. Medline

11. BrainyQuote. Margaret Meade quotation. Accessed March 1, 2020. https://www.brainyquote.com/quotes/margaret_mead_100502

12. McClelland S 3rd, Leberknight J, Guadagnolo BA, Coleman CN, Petereit DG. The pervasive crisis of diminishing radiation therapy access for vulnerable populations in the United States, part 2: American Indian patients. *Adv Radiat Oncol*. 2017 Aug 26;3(1):3-7. CrossRef. PMID 29556572.

13. Petereit DG, Guadagnolo BA, Wong R, Coleman CN. Addressing cancer disparities among American Indians through innovative technologies and patient navigation: The Walking Forward Experience. *Front Oncol*. 2011;1:11. CrossRef. PMID: 22649752

14. Holden DJ, Reiter K, O'Brien D, Dalton K. The strategic case for establishing public-private partnerships in cancer care. *Health Res Policy Syst*. 2015;13(1):44. CrossRef. Medline

15. Kaluzny AD, O'Brien DM. *Managing Disruptive Change in Healthcare: Lessons from a Public-Private Partnership to Advance Cancer Care and Research*. Oxford University Press; 2015:41-86.

16. Halpern MT, Spain P, Holden DJ, et al. Evaluation of the NCI's Community Cancer Centers' Program (NCCCP): impact on disparities in quality of cancer care. *J Health Dispar Res Pract*. 2015;8(1):63-80.
